# Validation of GWAS-Identified Variants for Anti-TNF Drug Response in Rheumatoid Arthritis: A Meta-Analysis of Two Large Cohorts

**DOI:** 10.3389/fimmu.2021.672255

**Published:** 2021-10-27

**Authors:** Jose Manuel Sánchez-Maldonado, Rafael Cáliz, Miguel Ángel López-Nevot, Antonio José Cabrera-Serrano, Ana Moñiz-Díez, Helena Canhão, Rob Ter Horst, Luca Quartuccio, Signe B. Sorensen, Bente Glintborg, Merete L. Hetland, Ileana Filipescu, Eva Pérez-Pampin, Pablo Conesa-Zamora, Jerzy Swierkot, Alfons A. den Broeder, Salvatore De Vita, Eva Rabing Brix Petersen, Yang Li, Miguel A. Ferrer, Alejandro Escudero, Mihai G. Netea, Marieke J. H. Coenen, Vibeke Andersen, João E. Fonseca, Manuel Jurado, Katarzyna Bogunia-Kubik, Eduardo Collantes, Juan Sainz

**Affiliations:** ^1^ Genomic Oncology Area, Centre for Genomics and Oncological Research (GENYO), Parque tecnológico de la Salud (PTS) Granada, Granada, Spain; ^2^ Hematology Department, Virgen de las Nieves University Hospital, Granada, Spain; ^3^ Instituto de Investigación Biosanitaria (IBs) Granada, Granada, Spain; ^4^ Department of Rheumatology, Virgen de las Nieves University Hospital, Granada, Spain; ^5^ Immunology Department, Virgen de las Nieves University Hospital, Granada, Spain; ^6^ EpiDoC Unit, CEDOC, NOVA Medical School and National School of Public Health, Universidade Nova de Lisboa, Lisbon, Portugal; ^7^ Comprehensive Health Research Center (CHRC), NOVA Medical School, Lisbon, Portugal; ^8^ Department of Internal Medicine and Radboud Center for Infectious Diseases, Radboud University Nijmegen Medical Center, Nijmegen, Netherlands; ^9^ Department of Medical Area, Clinic of Rheumatology, University of Udine, Udine, Italy; ^10^ Molecular Diagnostic and Clinical Research Unit, IRS-Center Sonderjylland, University Hospital of Southern Jutland, Aabenraa, Denmark; ^11^ Institute of Molecular Medicine, Faculty of Health Sciences, University of Southern Denmark, Odense, Denmark; ^12^ The Danish Rheumatologic Biobank and Copenhagen Center for Arthritis Research (DANBIO) Registry, The Danish Rheumatologic Biobank and Copenhagen Center for Arthritis Research (COPECARE), Center for Rheumatology and Spine Diseases, Centre of Head and Orthopaedics, Rigshospitalet, Glostrup, Denmark; ^13^ Department of Clinical Medicine, Faculty of Health and Medical Sciences, University of Copenhagen, Copenhagen, Denmark; ^14^ Rheumatology Department, University of Medicine and Pharmacy “Iuliu Hatieganu”, Cluj-Napoca, Romania; ^15^ Rheumatology Unit, University Hospital of Santiago de Compostela, Santiago de Compostela, Spain; ^16^ Clinical Analysis Department, Santa Lucía University Hospital, Cartagena, Spain; ^17^ Department of Rheumatology and Internal Medicine, Wroclaw Medical University, Wroclaw, Poland; ^18^ Radboud Institute for Health Sciences, Department of Rheumatology, Radboud University Medical Center, Nijmegen, Netherlands; ^19^ Department of Biochemistry and Immunology, University Hospital of Southern Jutland, Aabenraa, Denmark; ^20^ Centre for Individualised Infection Medicine (CiiM) & Centre for Experimental and Clinical Infection Research (TWINCORE), Helmholtz-Centre for Infection Research (HZI) and The Hannover Medical School (MHH), Hannover, Germany; ^21^ Rheumatology Department, Reina Sofía Hospital/Instituto Maimónides de Investigación Biomédica de Córdoba (IMIBIC)/University of Córdoba, Córdoba, Spain; ^22^ Department for Immunology & Metabolism, Life and Medical Sciences Institute (LIMES), University of Bonn, Bonn, Germany; ^23^ Radboud Institute for Health Sciences, Department of Human Genetics, Radboud University Medical Center, Nijmegen, Netherlands; ^24^ Institute of Regional Research, Faculty of Health Sciences, University of Southern Denmark, Odense, Denmark; ^25^ Rheumatology and Metabolic Bone Diseases Department, Hospital de Santa Maria, Centro Hospitalar Universitário Lisboa Norte (CHLN), Lisbon, Portugal; ^26^ Rheumatology Research Unit, Instituto de Medicina Molecular, Faculty of Medicine, University of Lisbon, Lisbon Academic Medical Center, Lisbon, Portugal; ^27^ Hirszfeld Institute of Immunology and Experimental Therapy, Polish Academy of Sciences, Wrocław, Poland; ^28^ Department of Biochemistry and Molecular Biology I, University of Granada, Granada, Spain

**Keywords:** GWAS, genetic variant, rheumatoid arthritis, drug response, TNF inhibitors

## Abstract

We aimed to validate the association of 28 GWAS-identified genetic variants for response to TNF inhibitors (TNFi) in a discovery cohort of 1361 rheumatoid arthritis (RA) patients monitored in routine care and ascertained through the REPAIR consortium and DANBIO registry. We genotyped selected markers and evaluated their association with response to TNFi after 6 months of treatment according to the change in disease activity score 28 (ΔDAS28). Next, we confirmed the most interesting results through meta-analysis of our data with those from the DREAM cohort that included 706 RA patients treated with TNFi. The meta-analysis of the discovery cohort and DREAM registry including 2067 RA patients revealed an overall association of the *LINC02549*_rs7767069_ SNP with a lower improvement in DAS28 that remained significant after correction for multiple testing (per-allele OR_Meta_=0.83, *P*_Meta_=0.000077; *P*_Het_=0.61). In addition, we found that each copy of the *LRRC55*_rs717117G_ allele was significantly associated with lower improvement in DAS28 in rheumatoid factor (RF)-positive patients (per-allele OR_Meta_=0.67, *P*=0.00058; *P*_Het_=0.06) whereas an opposite but not significant effect was detected in RF-negative subjects (per-allele OR_Meta_=1.38, *P*=0.10; *P*_Het_=0.45; *P*_Interaction_=0.00028). Interestingly, although the identified associations did not survive multiple testing correction, the meta-analysis also showed overall and RF-specific associations for the *MAFB*_rs6071980_ and *CNTN5*_rs1813443_ SNPs with decreased changes in DAS28 (per-allele OR_Meta_rs6071980_ = 0.85, *P*=0.0059; *P*_Het_=0.63 and OR_Meta_rs1813443_RF+_=0.81, *P*=0.0059; *P*_Het_=0.69 and OR_Meta_rs1813443_RF-_=1.00, *P*=0.99; *P*_Het_=0.12; *P*_Interaction_=0.032). Mechanistically, we found that subjects carrying the *LINC02549*_rs7767069T_ allele had significantly increased numbers of CD45RO+CD45RA+ T cells (*P*=0.000025) whereas carriers of the *LINC02549*_rs7767069T/T_ genotype showed significantly increased levels of soluble scavengers CD5 and CD6 in serum (*P*=0.00037 and *P*=0.00041). In addition, carriers of the *LRRC55*_rs717117G_ allele showed decreased production of IL6 after stimulation of PBMCs with *B burgdorferi* and *E coli* bacteria (*P*=0.00046 and *P*=0.00044), which suggested a reduced IL6-mediated anti-inflammatory effect of this marker to worsen the response to TNFi. In conclusion, this study confirmed the influence of the *LINC02549* and *LRRC55* loci to determine the response to TNFi in RA patients and suggested a weak effect of the *MAFB and CNTN5* loci that need to be further investigated.

## Introduction

Rheumatoid Arthritis (RA) is a complex and chronic disease marked by symptoms of inflammation and pain in the joints that eventually lead to joint destruction, loss of function and disability. These symptoms of inflammation are mostly driven by certain central cytokines that modulate both cellular and humoral immune responses in the synovial fluid and synovium of patients (1). Although RA remains as a chronic and incurable autoimmune disease that occurs in as much as 0.5-1% of the general population (2), the introduction of biological agents to target deregulated cytokines has substantially improved the signs and symptoms of the disease (3). Among these cytokines, tumor necrosis factor alpha (TNFα) has attracted most attention as it has been found to be deregulated in patients with autoimmune diseases including RA (4). It has been reported, for instance, that TNFα activates macrophages, synoviocytes, chondrocytes, and osteoclasts in a dose-dependent manner (5) and that high levels of circulating TNFα correlate with disease activity and disease progression (6).

The increasing number of biological agents approved by the FDA and the increased prevalence of the disease all around the world (7) have placed a substantial economic burden for health care systems. Although the introduction of biosimilars in clinical practice reduced the cost of these treatments in many countries (8), there is still an unmet need to optimize biologic therapies, avoiding unnecessary adverse effects risks and reducing costs (9).

The interplay between genetics and drug response has been the subject of intense investigations during last decades. Response to biologics has been shown to vary between individuals and that a large proportion of patients show no clinical improvement (10). Given the high cost of these drugs and the potential impairment of non-responding patients, the identification of genetic biomarkers associated with drug response to specific biological agents would help to know which patients might benefit from a particular treatment. However, to date, only a few genome-wide association studies (GWAS) (11–17) or well powered candidate gene association studies have been conducted (18–26). We are far from being able to optimize drug dosing or prioritize drug combinations based on genetic findings. In fact, attempts to validate the association of most of the genetic markers identified in these association studies have failed (27), which confirms the limited application of genetic findings in a clinical setting. Considering that the validation of previous GWAS findings is an essential step to tailor treatments for RA and to approach personalized medicine, we aimed to validate the association of GWAS-identified variants for response to TNF inhibitors (TNFi) in a two-stage nested case-control association study including a cohort of 1361 anti-TNF naïve RA patients ascertained through the REPAIR consortium and DANBIO registry and an independent replication cohort of 706 RA patients treated with TNFi from the DREAM registry. We also investigated whether the effect of selected markers on the response to TNFi could be modified by rheumatoid factor (RF) status, and whether genetic variants could influence immune responses and affect the serological concentration of 108 plasmatic inflammatory proteins, 7 serum steroid hormones or counts of 91 blood-derived immune cell populations.

## Material and Methods

### Study Populations and Response to TNFi

The discovery population consisted of 1361 RA patients ascertained through the REPAIR consortium and the DANBIO registry (**Table 1**) (28, 29). RA patients fulfilled the 1987 revised American College of Rheumatology (ACR) (30) and/or the ACR/EULAR 2010 classification criteria (31). In order to further replicate the most interesting results, we validated the association with response to anti-TNF drugs of those SNPs showing a *P*<0.05 in the discovery cohort in 706 Dutch RA patients treated with TNFi from the DREAM (Dutch RhEumatoid Arthritis Monitoring) registry (**Supplementary Table 1**). The study followed the Declaration of Helsinki. Study participants were of European origin and gave their written informed consent to participate in the study, which was approved by the ethical review committee of participant institutions: Virgen de las Nieves University Hospital (2012/89); Santa Maria Hospital-CHLN (CE 877/121.2012); University Clinical Hospital of Santiago de Compostela (2013/156); Wroclaw Medical University (KB-625/2016); Radboud university medical center (2011/299) and by the Regional Ethics Committee of Central Denmark Region (S-20120113). A detailed description of the discovery population has been reported elsewhere (19, 20, 22, 24). All RA patients were naïve for TNFi and response to TNFi for each patient in all study populations was calculated using the change in disease activity score (DAS28CRP) between baseline and 6 months after treatment. Overall and RF-stratified linear regression analyses adjusted for age, sex and country of origin were used to determine the association between GWAS-identified SNPs and changes in DAS28. RA patients with missing values either for DAS28 (in at the time points of interest) or RF were not included in the analysis.

**Table 1:** Demographic and clinical characteristics of anti-TNF patients.

Anti-TNF patients (n=1361)	
*Demographic characteristics*	*REPAIR consortium + DANBIO registry*
*Age (years)*	52 ± 14
*Sex ratio (female/male)*	3.4 (1050/310)
*Clinical assessment*	
*Percentage of patients with RF positivity* ^Ϯ^	721 (67.45)
*Percentage of ACPA-positive patients* ^ϕ^	728 (64.03)
*DAS28 at baseline*	5.91 ± 1.23
*Disease duration (years)*	12.92 ± 12.90
*Treatments*	
*First biologic agent*	
*Infliximab (%)*	386 (28.36)
*Etanercept (%)*	466 (34.24)
*Adalimumab (%)*	413 (30.35)
*Golimumab (%)*	48 (03.53)
*Certolizumab (%)*	67 (04.04)
*Biosimilars (%)*	16 (01.76)

Number of patients (%).

^Ϯ^RF status was available for 1069 patients.

^ϕ^ACPA status was available for 1137 patients.

### DNA Extraction, SNP Selection, Genotyping, and Quality Control

Genomic DNA from RA patients was extracted from blood samples using the QIAamp DNA Blood Mini kit (Valencia, CA, EEUU) according to manufacturer’s instructions. The single-nucleotide polymorphisms (SNPs) were selected through an extensive literature search of relevant GWAS and meta-analyses published by February 2019 using publicly available online databases. Additional criteria were potential functionality and linkage disequilibrium between the reported SNPs. Biological function was predicted according to the data publicly available in the integrated Regulome database (www.regulomedb.org), and eQTL data browsers (www.gtexportal.org/home/ and https://genenetwork.nl/bloodeqtlbrowser/). A total of 28 SNPs in 25 genes were selected for genotyping in the discovery cohort (**Table 2**). Genotyping of selected SNPs was performed using KASP^®^ probes according to manufacturer’s instructions (LGC Genomics, Hoddesdon, UK). For quality control, ~5% of DNA samples were randomly included as duplicates and concordance between duplicate samples was ≥99.0%. Replication of the most interesting association was conducted in the DREAM registry (n=706) following a similar quality control genotyping strategy.

**Table 2:** Selection of GWAS-identified SNPs for response to anti-TNF drugs.

dbSNP rs#	Chr.	Position (GRCh38.p7)	Nearest gene	Nucleotide substitution	Effect allele	SNP location	Reference
rs885813	1	21550581	ALPL	C/T	T	Intronic	(14)
rs885814	1	21549423	ALPL	C/T	T	Intronic	(14)
rs1813443	11	100140279	CNTN5	G/C	C	Intronic	(16)
rs8046065	16	3788297	CREBBP	C/T	T	Intronic	(14)
rs6138150	20	23866372	CST2||CST5	C/T	C	Intergenic	(15)
rs6028945	20	40192165	HSPEP1||MAFB	G/T	T	Intergenic	(15)
rs6071980	20	40239936	HSPEP1||MAFB	C/T	C	Intergenic	(15)
rs3849942	9	27543283	IFNK||C9orf72	T/C	T	ncRNA	(15)
rs13393173	2	168532581	LASS6	A/G	A	Intronic	(15)
rs4411591	18	6550118	LINC01387	C/T	A	Intronic	(16)
rs983332	1	87666697	LMO4||PKN2	A/C	A	Intergenic	(15)
rs1875620	9	88925144	C9orf47||LOC100128660||LOC100128911	A/G	A	Intergenic	(14)
rs1539909	18	71581359	CBLN2||LOC100132647	A/G	A	Intronic	(14)
rs7767069	6	68060671	LOC102723883||LINCO2549	A/T	T	Downstream	(16)
rs1568885	7	13597906	LOC107986770||ETV1	A/T	T	Intronic	(16)
rs10520789	15	95598638	LINC00924	A/G	A	Downstream	(14)
rs11870477	17	69806211	MAP2K6||KCNJ16	A/C	C	Intronic	(14)
rs2378945	14	31831584	NUBPL	G/A	A	Intronic	(16)
rs717117	11	57127131	OR5BP1P||LRRC55	A/G	G	Intronic	(14)
rs1532269	5	32018735	PDZD2	C/G	G	Intronic	(17)
rs4651370	1	187269960	PLA2G4A||FDPSL1	A/T	A	Intronic	(16)
rs854547	7	95294544	PPP1R9A	A/G	G	3’UTR	(15)
rs854548	7	95296508	PPP1R9A||PON1	A/G	A	Downstream	(15)
rs10945919	6	163765645	QKI||LOC728275	A/G	G	Intronic	(15)
rs437943	4	35370476	CENTD1	A/G	G	Upstream	(15)
rs3794271	12	20707159	SLCO1C1	C/T	C	Intronic	(14)
rs4694890	4	48224250	TEC	A/C	C	Intronic	(17)
rs1447722	3	139835611	TRMT112P5	C/G	C	Intergenic	(16)

SNP, single nucleotide polymorphism; MAF, minor allele frequency; UTR, untranslated region.

References: (14–17).

### Hardy-Weinberg Equilibrium, Genetic Association Analysis, and Meta-Analysis

Deviation from Hardy-Weinberg Equilibrium (HWE) was tested in the control group (responders and moderate responders according to the EULAR response criteria) by chi-square (χ2), and linear regression analysis adjusted for age, sex and country of origin was used to assess the associations of the GWAS-identified polymorphisms with absolute changes in DAS28 assuming log-additive, dominant and recessive models of inheritance. Those SNPs with the lowest P-value in the discovery population according to each genetic model were advanced for replication in the DREAM cohort and meta-analysis of the discovery and replication populations using a fixed effect model was performed to validate the association observed. I2 statistic was used to assess heterogeneity between studies. Correction for multiple testing was performed using the Bonferroni method but also considering the two inheritance models tested. Given that log-additive and dominant models showed a high degree of collinearity, the significant threshold for the meta-analysis was set to 0.00089 considering log-additive/dominant and recessive inheritance models (0.05/28SNPs/2models). Overall statistical power was calculated using Quanto (v.12.4) assuming a log-additive model and a baseline risk of 30% for response to TNFi (32, 33).

### Cell Isolation, Differentiation and Cytokine Quantitative Trait Loci, and Hormone Analysis in Relation to the GWAS-Identified Variants for Response To TNFi

With the aim of determining whether those SNPs associated with response to TNFi had a role in modulating immune responses, we performed *in vitro* stimulation experiments and measured cytokine production (IFNγ, IL1Ra, IL1β, IL6, IL8, IL10, TNFα, IL17, and IL22) after stimulation of peripheral blood mononuclear cells (PBMCs), whole blood or monocyte-derived macrophages (MDMs) from 408 healthy subjects of the 500FG cohort from the Human Functional Genomics Project (HFGP) with LPS (1 or 100 ng/ml), PHA (10μg/ml), Pam3Cys (10μg/ml), CpG (ODN M362; 10μg/ml) and *B. burgdorferi* and *E. coli*, as experimental model for cytokine production capacity. Given the sex disparities in the prevalence and course of RA and the impact of steroid hormones in modulating immune responses, we also evaluated the correlation of SNPs with serum levels of 7 steroid hormones (androstenedione, cortisol, 11-deoxy-cortisol, 17-hydroxy progesterone, progesterone, testosterone and 25 hydroxy vitamin D3) in a subset of the 500FG cohort without hormonal replacement therapy or oral contraceptives (n=280). After log transformation, cytokine or serum steroid hormone levels were correlated with the SNPs of interest using a linear regression model with age and sex as co-factors in R (http://www.r-project.org/). This analysis led to cytokine quantitative trait loci (cQTL) and hormone quantitative trait loci (hQTL). Significance thresholds were set to be 0.000463 and 0.00357 (0.05/6stimulants/9cytokines or 0.05/7hormones and 2 inheritance models) for cQTL and hQTL, respectively.

### Correlation Between GWAS-Identified Polymorphisms and Cell Counts of 91 Blood-Derived Immune Cell Populations and Serum/Plasmatic Proteomic Profile

We also investigated whether selected polymorphisms had an impact on blood cell counts by analyzing a set of 91 manually annotated immune cell populations and genotype data from the 500FG cohort that consisted of 408 healthy subjects (**Supplementary Table 2**). Cell populations were measured by 10-color flow cytometry (Navios flow cytometer, Beckman Coulter) after blood sampling (2-3 hours) and cell count analysis was performed using the Kaluza software (Beckman Coulter, v.1.3). In order to reduce inter-experimental noise and increase statistical power, cell count analysis was performed by calculating parental and grandparental percentages, which were defined as the percentage of a certain cell type within the cell-populations one or two levels higher in the hierarchical definitions of cell sub-populations (34). Detailed laboratory protocols for cell isolation, reagents, gating and flow cytometry analysis have been reported elsewhere (35) and the accession number for the raw flow cytometry data and analyzed data files are available upon request to the authors (http://hfgp.bbmri.nl). A proteomic analysis was also performed in serum and plasma samples from the 500FG cohort. Circulating proteins were measured using the commercially Olink^®^ Inflammation panel (Olink, Sweden) that resulted in the measurement of 103 different biomarkers (**Supplementary Table 3**). Proteins levels were expressed on a log2-scale as normalized protein expression values, and normalized using bridging samples to correct for batch variation. Considering the number of proteins (n=103) and cell populations (n=91) tested, *P*-values of 0.00049 and 0.00055 were set as significant thresholds for the proteomic and cell-level variation analysis, respectively.

## Results

A total of 1361 anti-TNF patients were included in the discovery population. The mean age of the RA patients was 52±14 and they showed a female/male ratio of 3.4 (1050/310). Sixty-seven percent of the RA patients were positive for RF and 64% had anti-citrullinated protein antibodies (ACPA). The median disease duration was of 12.92 years and the disease activity score 28 (DAS28CRP) calculated at patient recruitment was of 5.91 (**Table 1**).

### Association of GWAS-Identified SNPs With Response to Anti-TNF Drugs

All SNPs were in Hardy-Weinberg equilibrium in the control group (responders according to EULAR response criteria; *P*>0.001) and showed a high genotyping call rate (>90%) with the exception of the *LINC01387*_rs4411591_ SNP that was excluded from the statistical analysis. The overall linear regression analysis of the discovery cohort including 1361 RA patients treated with TNFi showed that the *MAFB*_rs6028945_, *MAFB*_rs6071980_, *LINC02549*_rs7767069_, and *LRRC55*_rs717117_ SNPs had an overall significant effect on the response to TNFi at P<0.05 level (OR_Dominant_=0.81, 95%CI 0.68-0.97, *P*=0.020; per-allele OR=0.83, 95%CI 0.72-0.97, *P*=0.020; per-allele OR=0.85, 95%CI 0.76-0.96, *P*=0.008; per-allele OR=0.76, 95%CI 0.60-0.97, *P*=0.026; **Table 3**). Importantly, the meta-analysis of the discovery and replication cohorts confirmed the overall association of the *LINC02549*_rs7767069_ SNP with lower DAS28 improvement that remained significant after multiple testing correction (per-allele OR_Meta_=0.83, 95%CI 0.76-0.91, *P*_Meta_=0.000077; *P*_Het_=0.61; **Table 4**). Although it did not survive multiple testing correction, the meta-analysis also showed a potentially interesting overall associations for the *MAFB*_rs6071980_ SNP with less DAS28 improvement (per-allele OR_Meta_rs6071980 =_ 0.85, 95%CI 0.76-0.95, *P*=0.0059; *P*_Het_=0.63; **Table 4**).

**Table 3:** Overall and RF-specific associations of selected polymorphisms and response to anti-TNF drugs (ΔDAS28) in the REPAIR consortium.

Gene	SNP ID	Effect allele	Overall		RF-positive patients		RF-negative patients		*P_Interaction_*
			REPAIR+DANBIO (n=1361)		REPAIR+DANBIO (n=721)		REPAIR+DANBIO (n=347)		
			OR (95% CI)^δ^	*P*	OR (95% CI)^δ^	*P*	OR (95% CI)^δ^	*P*	
ALPL	rs885813	T	0.96 (0.86-1.07)	0.48	1.06 (0.91-1.22)	0.47	**0.80 (0.65-0.99)**	**0.040**	0.13
ALPL	rs885814	T	0.96 (0.85-1.07)	0.45	0.91 (0.78-1.06)	0.24	1.18 (0.95-1.47)	0.14	0.08
CNTN5	rs1813443	C	0.97 (0.84-1.13)^†^	0.72	**0.79 (0.65-0.97)^†^**	**0.023**	1.14 (0.86-1.53)^†^	0.34	**0.032**
CREBBP	rs8046065	T	1.01 (0.87-1.19)	0.85	1.18 (0.96-1.45)	0.11	0.82 (0.60-1.10)	0.19	0.07
CST2||CST5	rs6138150	C	1.06 (0.92-1.22)	0.40	1.04 (0.87-1.26)	0.66	1.01 (0.77-1.32)	0.96	0.72
HSPEP1||MAFB	rs6028945	T	**0.81 (0.68-0.97)^†^**	**0.020**	0.89 (0.70-1.21)^†^	0.31	0.78 (0.53-1.09)^†^	0.14	0.57
HSPEP1||MAFB	rs6071980	C	**0.83 (0.72-0.97)**	**0.020**	0.85 (0.70-1.03)	0.10	0.81 (0.58-1.14)	0.23	0.73
IFNK||C9orf72	rs3849942	T	1.02 (0.90-1.15)	0.74	0.93 (0.79-1.10)	0.39	1.21 (0.95-1.55)	0.13	0.27
LASS6	rs13393173	A	1.10 (0.96-1.25)	0.19	1.08 (0.91-1.28)	0.41	1.18 (0.89-1.55)	0.25	0.48
LMO4||PKN2	rs983332	A	1.12 (0.97-1.29)	0.11	1.14 (0.95-1.38)	0.16	1.07 (0.84-1.38)	0.58	0.68
C9orf47	rs1875620	A	0.94 (0.84-104)	0.24	0.93 (0.81-1.08)	0.34	**0.80 (0.65-0.99)**	**0.037**	0.29
CBLN2|| LOC100132647	rs1539909	A	0.96 (0.81-1.13)	0.62	0.88 (0.70-1.10)	0.27	0.84 (0.61-1.16)	0.29	1.00
LOC102723883||LINC02549	rs7767069	T	**0.85 (0.76-0.96)**	**0.008**	0.91 (0.78-1.08)	0.28	0.81 (0.65-1.01)	0.058	0.11
LOC107986770||ETV1	rs1568885	T	0.67 (0.40-1.10)^§^	0.11	0.84 (0.42-1.66)^§^	0.61	**0.44 (0.20-0.98)^§^**	**0.046**	0.23
LOC400456||LOC100132798	rs10520789	A	1.03 (0.86-1.22)	0.76	1.14 (0.90-1.44)	0.29	1.01 (0.72-1.41)	0.97	0.48
MAP2K6||KCNJ16	rs11870477	C	1.07 (0.91-1.25)	0.42	0.92 (0.74-1.14)	0.46	1.18 (0.85-1.65)	0.33	0.13
NUBPL	rs2378945	A	1.02 (0.91-1.13)	0.79	1.06 (0.91-1.24)	0.44	0.97 (0.79-1.20)	0.81	0.41
OR5BP1P||LRRC55	rs717117	G	**0.76 (0.60-0.97)**	**0.026**	**0.54 (0.39-0.74)**	**0.00012**	1.52 (0.96-2.42)	0.07	**0.00028**
PDZD2	rs1532269	G	1.01 (0.88-1.16)	0.86	1.03 (0.89-1.20)	0.68	1.02 (0.82-1.26)	0.87	0.95
PLA2G4A||FDPSL1	rs4651370	A	1.05 (0.90-1.23)	0.53	1.09 (0.88-1.35)	0.42	0.92 (0.69-1.24)	0.60	0.15
PPP1R9A	rs854547	G	1.00 (0.89-1.11)	0.95	1.03 (0.89-1.19)	0.71	0.92 (0.74-1.14)	0.43	0.46
PPP1R9A||PON1	rs854548	A	1.01 (0.89-1.15)	0.89	1.04 (0.87-1.24)	0.67	0.85 (0.66-1.10)	0.22	0.39
QKI||LOC728275	rs10945919	G	0.88 (0.76-1.03)^†^	0.11	**0.79 (0.64-0.97)^†^**	**0.027**	1.03 (0.77-1.38)^†^	0.85	0.14
SEC63P3	rs437943	G	1.10 (0.95-1.28)^†^	0.21	0.98 (0.80-1.20)^†^	0.82	**1.37 (1.02-1.85)^†^**	**0.037**	0.25
SLCO1C1	rs3794271	C	0.95 (0.85-1.07)	0.39	0.96 (0.82-1.11)	0.57	1.02 (0.82-1.27)	0.87	0.34
TEC	rs4694890	C	0.96 (0.86-1.07)	0.42	1.01 (0.87-1.17)	0.86	0.84 (0.67-1.05)	0.12	0.37
TRMT112P5	rs1447722	C	1.03 (0.92-1.15)	0.61	1.01 (0.87-1.18)	0.85	1.08 (0.87-1.35)	0.48	0.81

SNP, single nucleotide polymorphism; OR, odds ratio; CI, confidence interval.

Data on RF was available in 1069 RA patients. Estimates were adjusted for age, sex and country of origin. P < 0.05 in bold.

^δ^Estimates calculated according to a log-additive model of inheritance.

^†^Estimates calculated according to a dominant model of inheritance.

**^§^** Estimates calculated according to a recessive model of inheritance.

**Table 4:** Overall and RF-specific meta-analysis of the *CNTN5*_rs1813443_ *, MAFB*_rs607198_ *, LINCO2549*_rs7767069_ and *LRRC55*_rs717117_ polymorphisms and response to anti-TNF drugs.

Gene	SNP ID	Effect allele	Overall		Overall		Meta-analysis (n=2067)		*P_Heterogeneity_*
			REPAIR+DANBIO (n=1361)		DREAM (n=706)			
			OR (95% CI)^δ^	*P*	OR (95% CI)^δ^	*P*	OR (95% CI)^δ^	*P*
CNTN5	rs1813443	C	0.97 (0.84-1.13)^†^	0.72	**0.82 (0.68-1.00)^†^**	**0.046**	0.91 (0.81-1.03)^†^	0.12	0.18
HSPEP1||MAFB	rs6071980	C	**0.83 (0.72-0.97)**	**0.020**	0.88 (0.73-1.06)	0.18	**0.85 (0.76-0.95)**	**0.0059**	0.63
LOC102723883||LINC02549	rs7767069	T	**0.85 (0.76-0.96)**	**0.008**	**0.80 (0.70-0.93)**	**0.004**	**0.83 (0.76-0.91)**	**0.00007**	0.61
OR5BP1P||LRRC55	rs717117	G	**0.76 (0.60-0.97)**	**0.026**	0.89 (0.67-1.20)	0.46	**0.81 (0.67-0.98)**	**0.026**	0.41
		**Effect allele**	**RF-positive patients**		**RF-positive patients**		**RF-positive patients**		
			**REPAIR+DANBIO (n=721)**		**DREAM (n=532)**		**Meta-analysis (n=1253)**		
**Gene**	**SNP ID**		**OR (95% CI)** ^δ^	***P***	**OR (95% CI)** ^δ^	***P***	**OR (95% CI)** ^δ^	***P***	***P_Heterogeneity_***
CNTN5	rs1813443	C	**0.79 (0.65-0.97)^†^**	**0.023**	0.84 (0.67-1.04)^†^	0.12	**0.81 (0.70-0.94)^†^**	**0.0059**	0.69
HSPEP1||MAFB	rs6071980	C	0.85 (0.70-1.03)	0.10	0.85 (0.69-1.04)	0.11	**0.85 (0.73-0.98)**	**0.023**	1.00
LOC102723883||LINC02549	rs7767069	T	0.91 (0.78-1.08)	0.28	**0.82 (0.69-0.97)**	**0.022**	**0.87 (0.77-0.97)**	**0.016**	0.39
OR5BP1P||LRRC55	rs717117	G	**0.54 (0.39-0.74)**	**0.00012**	0.83 (0.60-1.15)	0.28	**0.67 (0.54-0.84)**	**0.00058**	0.06
		**Effect allele**	**RF-negative patients**		**RF-negative patients**		**RF-positive patients**		
			**REPAIR+DANBIO (n=347)**		**DREAM (n=154)**		**Meta-analysis (n=501)**		
**Gene**	**SNP ID**		**OR (95% CI)** ^δ^	***P***	**OR (95% CI)** ^δ^	***P***	**OR (95% CI)** ^δ^	***P***	***P_Heterogeneity_***
CNTN5	rs1813443	C	1.14 (0.86-1.53)^†^	0.34	0.76 (0.50-1.16)^†^	0.20	1.00 (0.79-1.27)^†^	0.99	0.12
HSPEP1||MAFB	rs6071980	C	0.81 (0.58-1.14)	0.23	1.10 (0.71-1.71)	0.67	0.91 (0.69-1.19)	0.48	0.28
LOC102723883||LINC02549	rs7767069	T	0.81 (0.65-1.01)	0.058	0.76 (0.55-1.04)	0.08	**0.79 (0.66-0.95)**	**0.012**	0.75
OR5BP1P||LRRC55	rs717117	G	1.52 (0.96-2.42)	0.07	1.10 (0.55-2.19)	0.80	1.38 (0.94-2.02)	0.10	0.45

SNP, single nucleotide polymorphism; OR, odds ratio; CI, confidence interval.

Response to anti-TNF defined as ΔDAS28. Data on RF was available in 1068 and RA patients in the discovery (REPAIR+DANBIO) and replication cohorts (DREAM).

Association estimates were adjusted for age, sex and country of origin in the discovery cohort and age and sex in the replication cohort (all Dutch patients). P < 0.05 in bold.

^δ^Estimates calculated according to a log-additive model of inheritance.

^†^Estimates calculated according to a dominant model of inheritance.

A RF-stratified analysis showed a RF-specific association for the *LRRC55*_rs717117_ SNP with response to TNFi that remained statistically significant after correction for multiple testing in the discovery population. Thus, RF-positive RA patients carrying the *LRRC55*_rs717117G_ allele additively decreased the drop in DAS28 (per-allele OR=0.54, 95%CI 0.39–0.74, *P*=0.00012) whereas RF-negative RA patients showed an opposite but not statistically significant effect (per-allele OR=1.52, 95%CI 0.96–2.42, *P*=0.07; *P*_Interaction_=0.00028; **Table 3**). Interestingly, the meta-analysis of our data with those from the DREAM registry including 2067 RA patients confirmed the RF-specific effect of this SNP to modulate the response to anti-TNF drugs (per-allele OR_Meta_RF+_=0.67, 95%CI 0.54-0.84, *P*_Meta_=0.00058; *P*_Het_=0.06 and per-allele OR_Meta_RF-_=1.38, 95%CI 0.94-2.02, *P*=0.10; *P*_Het_=0.45; *P*_Interaction_=0.00028; **Table 4**). Although it did not survive multiple testing, the meta-analysis also showed potentially interesting RF-specific association for the *CNTN5*_rs1813443_ SNP with a decreased drop in DAS28 (OR_Meta_rs1813443_RF+_=0.81, 95%CI 0.70-0.94, *P*=0.0059; *P*_Het_=0.69 and OR_Meta_rs1813443_RF-_=1.00, 95%CI 0.79-1.27, *P*=0.99; *P*_Het_=0.12; *P*_Interaction_=0.032; **Table 4**).

### Functional Characterization of the Most Interesting Findings

**Figure 1:**
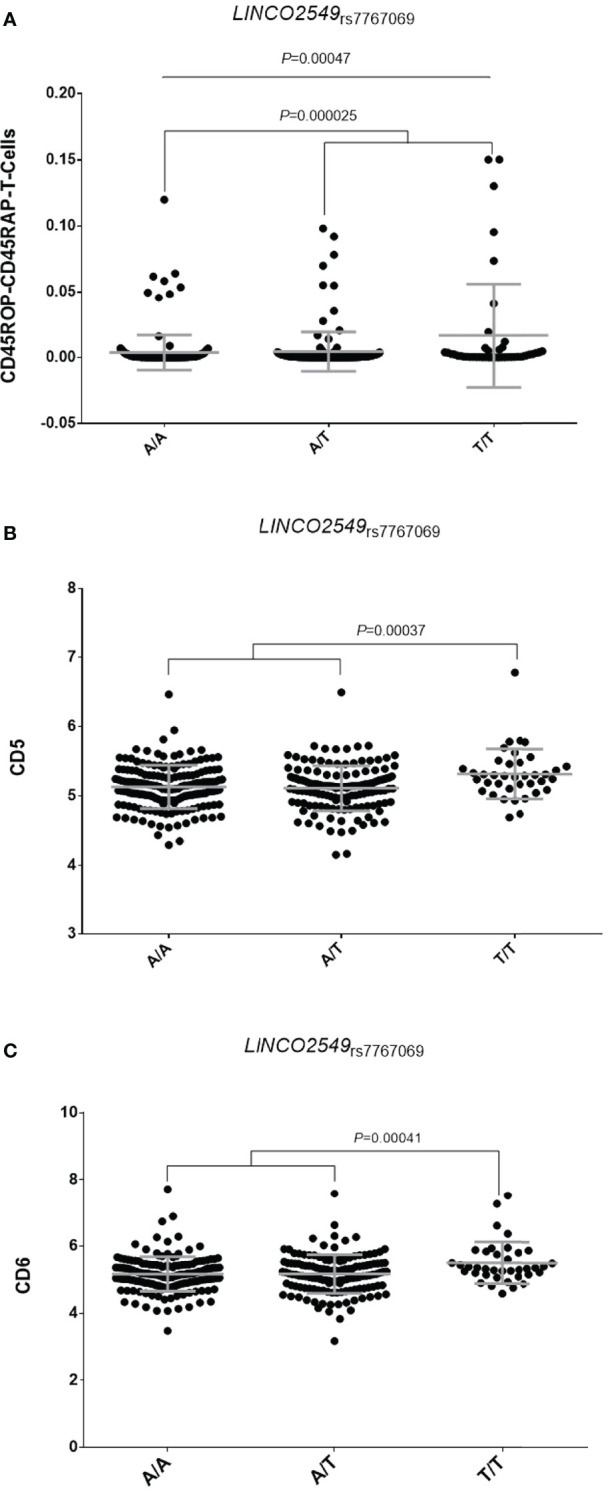
Correlation of the LINC02549_rs7767069_ polymorphism with absolute numbers of CD45RO+CD45RA+ T cells in blood **(A)** and serum levels of soluble scavenger receptors CD5 **(B)** and CD6 **(C)**.

Considering these results, we attempted to shed some light into the functional consequences of the overall or RF-specific effects of the *LINC02549*_rs7767069_, *LRRC55*_rs717117_, *MAFB*_rs6071980_ and *CNTN5*_rs1813443_ SNPs to modulate the response to TNFi. Interestingly, our functional experiments showed that, when considering the total number of leukocytes as reference, the *LINC02549*_rs7767069_ polymorphism significantly correlated with increased numbers of CD45RO+CD45RA+ T cells in blood (*P*=0.00047; **Figure 1A**). Subjects carrying the *LINC02549*_rs7767069T_ allele (associated with poor response to TNFi in RA patients) had significantly increased numbers of CD45RO+CD45RA+ T cells (*P*=0.000025), which suggested that this genetic marker might influence the response to TNFi by mediating the number of this specific T cell subset in blood and, thereby contribute to inflammation. In addition, we observed that those subjects carrying two copies of the *LINC02549*_rs7767069T_ allele showed significantly increased serum levels of soluble scavenger receptors CD5 and CD6 when compared with those carrying the A/T or A/A genotypes (*P*=0.00037 and *P*=0.00041; **Figures 1B, C**). These results also suggested a functional role of the *LINC02549*_rs7767069_ SNP in RA likely through the CD5/CD6-mediated modulation of T cells and certain subsets of B cells that control multiple processes including cellular adhesion and migration across endothelial and epithelial cells, antigen presentation by B cells and the subsequent proliferation of T cells.

**Figure 2:**
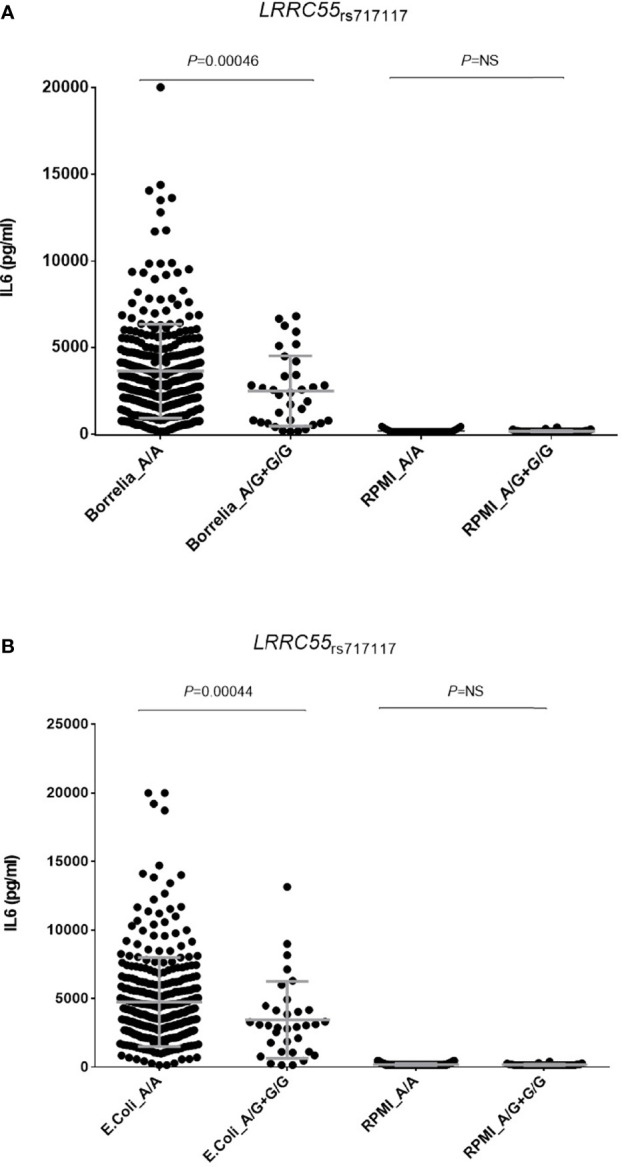
Correlation of the LRRC55_rs717117G_ allele and levels of IL6 after stimulation of PBMCs either with *B. burgdorferi* **(A)** or *E. coli* **(B)**.

**Figure 3:**
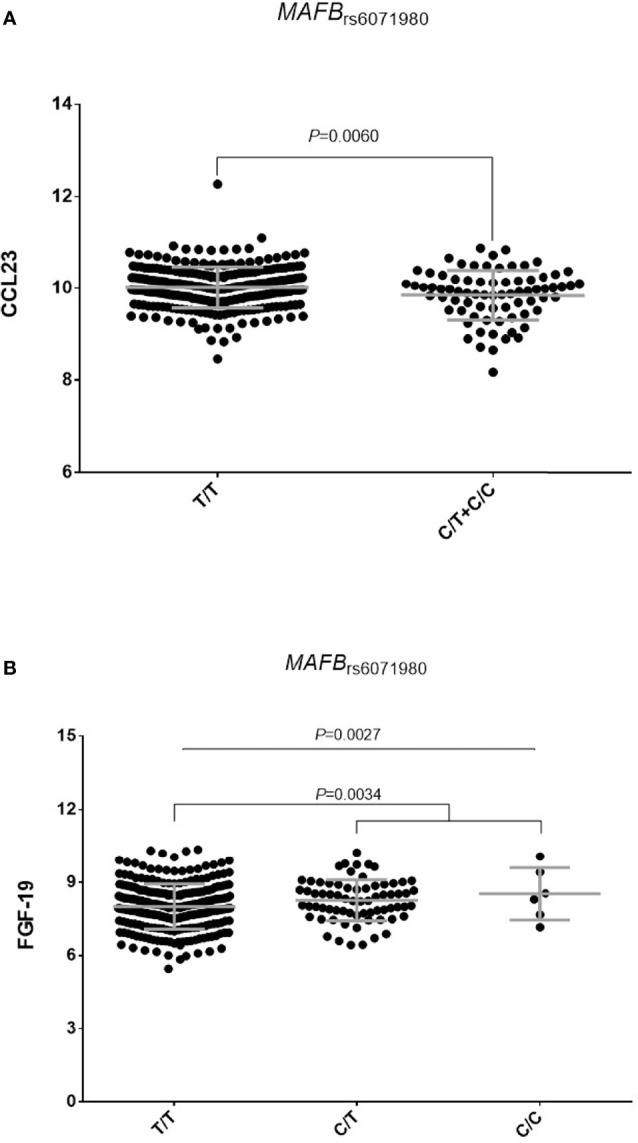
orrelation of the MAFB_rs6071980C_ allele with serum levels of CCL23 **(A)** and FGF-19 proteins **(B)**.

Furthermore, although we could not stratify our functional analyses by RF status because of the healthy nature of the blood donors, we found that carriers of the *LRRC55*_rs717117G_ allele showed significantly decreased levels of IL6 production after stimulation of PBMCs with either *B. burgdorferi* (*P*=0.00046; **Figure 2A**) or *E. coli* (*P*=0.00044; **Figure 2B**), which suggested an implication of the *LRRC55* locus in the modulation of the response to TNFi by regulating IL6 production and likely IL6-mediated T cell differentiation into effector Th2 cells. Functional data from Haploreg also showed that the *LRRC55*_rs717117_ variant correlates with mRNA *P2RX3* expression levels, a well-known gene involved in controlling T cell proliferation. Finally, our functional experiments revealed that carriers of the *MAFB*_rs6071980_^C^ allele showed decreased levels of Chemokine (C-C motif) ligand 23 (CCL23; *P*=0.0060; **Figure 3A**) and increased levels of serum Fibroblast growth factor 19 (FGF-19; *P*=0.0034; **Figure 3B**). Whereas FGF-19 protein modulates inflammation by mediating IL6 production, CCL23 has been implicated in monocyte recruitment during inflammation and it has been previously shown to positively correlate with drop in DAS28 after treatment with TNFi. Although the effect of the *MAFB* SNP to modulate either serum FGF-19 or CCL23 levels did not remain significant after correction for multiple testing, these results might indicate a weak, but still functional, effect of the *MAFB* locus in modulating response to anti-TNF drugs.

## Discussion

Although treatment of RA patients using monoclonal anti-TNF drugs has been a particularly successful approach to control inflammation and to prevent joint destruction and the appearance of bone erosions, non-responsiveness is prevalent and no effective biomarkers for drug response prediction have been consistently identified (36). This comprehensive validation study aimed at confirming the association of GWAS-identified variants with response to TNFi and to shed some light into the biological mechanisms underlying the most interesting associations. For that purpose, we conducted a two-stage case control study including 2067 RA patients treated with anti-TNF drugs ascertained through the REPAIR consortium but also DANBIO and DREAM registries. The most significant result was the overall association of the *LINC02549*_rs7767069_ SNP with a poor response to anti-TNF drugs. The meta-analysis of the discovery and replication cohorts showed that each copy of the *LINC02549*_rs7767069T_ allele significantly decreased the improvement in DAS28 by 17% after the treatment with a TNFi. Importantly, the association of the *LINC02549*_rs7767069_ variant with poor response to TNFi was significant in the two populations analyzed and remained significant after correction for multiple testing, which confirmed a role of the *LINC02549* locus in the modulation of response to anti-TNF drugs.

*LINC02549* (Long Intergenic Protein Coding RNA 2549) is an RNA gene that is affiliated with the lncRNA class, which represents a large proportion of the human transcriptome. *LINC02549* maps to chromosome 6 and it is expressed in resting T cells and CD4 activated T cells. Although its function is still largely unknown, our data suggest that it might exert a role in determining the number of circulating CD45RO+CD45RA+ T cells, which are a subset of cells frequently found in the synovial fluid of both chronic arthritis (37) and RA patients (38). According to the results of Koch et al. (1990), CD45RA+ CD45RO+ T lymphocytes are mostly detected in perivascular regions, which suggest that these lymphocytes might access the RA synovial tissue *via* the synovial vasculature (38) and that, once there, they could play a role in promoting synovial tissue inflammation mainly by the induction of memory immune responses. Therefore, it seems to be plausible to suggest that the negative impact of the *LINC02549*_rs7767069_ SNP on the response to TNFi might be mediated by its role in modulating numbers of CD45RA+CD45RO+ T lymphocytes that could migrate to the synovial tissue and promote inflammatory responses and, thereby hamper the control of inflammation during treatment with anti-TNF drugs. In support of this hypothesis, we found that carriers of two copies of the *LINC02549*_rs7767069T_ allele also showed significantly increased levels of soluble scavenger receptors CD5 and CD6 (sCD5 and sCD6) in serum that are proteins highly expressed in regulatory T cells and a specific subset of B cells (CD5+ or B1a) (39). Although the origin of these soluble scavenger receptors in RA is poorly understood, it has been suggested that they are shed in the serum by proteases from the surface of activated lymphocytes that subsequently infiltrate synovium structures (40). In fact, increased serum levels of sCD5 and sCD6 has been found in subjects diagnosed with RA (41–44) but also other autoimmune diseases such as primary Sjögren’s syndrome (42, 45, 46), systemic inflammatory response syndrome (47), multiple sclerosis (44) or dermatitis (48). Although the functional role of both soluble scavengers in autoimmune diseases is still under investigation, it is well established that sCD5 and sCD6 are regulators of T cell functions and induce autoreactivity. It is known that they are required for the initiation, differentiation and maintenance of T cell immune responses (49, 50) but also T cell migration and extravasation to the synovial tissue (51). Furthermore, it has been reported that both sCD5 and sCD6 are involved in the modulation of TCR and BCR signaling and determinate T- and B-cell survival (52) and Th17 differentiation (53, 54). Furthermore, clinical trials using humanized anti-CD6 mAbs have provided valuable information regarding the potential targeting of CD6 for the treatment of RA but also psoriasis and potentially other T cell–driven autoimmune diseases (55–57). Recent investigations have also suggested that genetic alterations within the CD6 gene associated with clinical outcome of several autoimmune diseases (44, 58) and correlated with the response to TNFi (59), which pointed to a role of these soluble scavenger receptors in modulating response to anti-TNF drugs. Considering these findings, we hypothesize that, besides its effect on modulating number of the CD45RA+CD45RO+ T lymphocytes, the *LINC02549*_rs7767069_ SNP might negatively influence the response to anti-TNF drugs by stimulating directly or indirectly the production of sCD5 and sCD6 and thereby inducing long-term T cell-mediated immune responses.

Another interesting result that remained significant after correction for multiple testing was the RF-specific association of the *LRRC55*_rs717117_ SNP with lower changes in DAS28 after the treatment with TNFi. The meta-analysis of the discovery and replication cohorts showed that RF-positive patients carrying the *LRRC55*_rs717117G_ allele have a significantly decreased drop in DAS28 after treatment with a TNFi, whereas an opposite but not statistically significant effect was observed in RF-negative RA patients. Noticeably, functional experiments showed that, after stimulation of PBMCs from healthy subjects with *B. burgdorferi* and *E. coli* bacteria, carriers of the *LRRC55*_rs717117G_ allele showed significantly decreased production of IL6 when compared to those carrying the most common genotype. Although functional experiments could not be stratified by RF because of the healthy nature of blood donors, these results suggested a role of the *LRRC55* locus in modulating IL6-mediated immune responses. On the other hand, functional data from Haploreg also suggested an implication of the *LRRC55*_rs717117_ variant in controlling *P2RX3*-mediated T cell proliferation.

*LRRC55* gene maps on chromosome 11 and it encodes for the leucine-rich repeat-containing protein 55, a protein that belongs to the LRRC superfamily that include hundreds of proteins mainly expressed in brain. Several LRRC proteins have been linked to the regulation of ion channels (60) but it has been also demonstrated that LRRC proteins are also implicated in modulating immune responses against bacterial pathogens (61) and modulate cell trafficking of membrane receptors such as toll-like receptors (62). Although the interplay between LRRC55 and IL6 has not been demonstrated, our experimental data suggest that the *LRRC55*_rs717117_ SNP modulates IL6 production in response to bacteria and, therefore, might be involved in other IL6-dependent immune processes that could worsen the response to TNFi.

It is widely known that IL6 can induce both anti-inflammatory and pro-inflammatory immune responses, which depend entirely on the signalling pathway triggered. Whereas anti-inflammatory responses are mostly mediated by the classic signalling cascade (through binding to the transmembrane IL6 receptor), pro-inflammatory responses and chronic inflammation are mediated by trans-signalling (through binding to the soluble IL6 receptor) or by the interaction of IL6R with gp130 (63, 64). Considering our functional data, it is conceivable to suggest that the *LRRC55*_rs717117_ SNP might affect the response to TNFi by decreasing IL6 production and thus inhibiting the classical IL6-dependent anti-inflammatory pathway and dysregulating pro-inflammatory responses. In support of this hypothesis, several mouse models have shown that the activation of the IL6 classic signaling pathway is essential for the activation of STAT3-mediated signaling pathways which reduce inflammation and induce the regeneration of the affected tissues (65). In addition, it has been reported that IL6 is one of the earliest factors that trigger the differentiation of naive T cells into effector Th2 cells *in vitro* and that, when absent, aggravates the development of the inflammatory processes (64).

Finally, although the genetic association of the *MAFB*_rs6071980_ SNP with lower response to TNFi did not remain significant after correction for multiple testing, we found that the *MAFB*_rs6071980_ SNP correlated with higher levels of serum FGF-19 and decreased levels of CCL23. Given that FGF-19 is a master protein involved in the inhibition of intestinal inflammation (66, 67) and CCL23 has been positively correlated with the DAS28 score in RA patients (68), we think that it would worth to investigate more in detail the impact of this SNP on drug response in future studies. In addition, it might be interesting to further analyze the weak association of the *CNTN5*_rs1813443_ SNP with poor response to TNFi. However, given that we could not find any significant impact of this marker on immune responses, blood cell counts or serum inflammatory proteins or steroid hormones, we are prone to think that this SNP might not have a relevant role in modulating response to TNFi.

This study has both strengths and weaknesses. Among the strengths we can highlight the use of large and well-characterized RA patient populations that allowed the development of a well-powered overall association analysis but also to investigate the effect modification by RF status. Overall, we had 80% of power to detect an OR of 1.18 (α=0.00089) for a SNP with a frequency of 0.25. On the other hand, it is worth mentioning the comprehensive analysis of the functional effect of the most interesting genetic variants on modulating immune responses, which was performed using a large sample size for this kind of studies. We analysed cQTL and hQTL data but also counts of 91 blood-derived cell populations and serum levels of 103 immunological proteins. An important limitation of this study was the impossibility to adjust linear regression analyses for potential confounding factors including concomitant treatments that might influence the response to TNFi. In addition, given the healthy nature of the subjects included in the HFGP cohort, we could not control our functional experiments by RF status.

In conclusion, this study validates the overall or RF-specific association of *LINC02549* and *LRRC55* loci with the response to TNFi and provides new insights into the functional role of these polymorphisms in modulating immune responses and response to anti-TNF drugs.

## Data Availability Statement

All data used in this project have been meticulously catalogued and archived in the BBMRI-NL data infrastructure (https://hfgp.bbmri.nl/) using the MOLGENIS open-source platform for scientific data (69). This allows flexible data querying and download, including sufficiently rich metadata and interfaces for machine processing (R statistics, REST API) and using FAIR principles to optimize Findability, Accessibility, Interoperability and Reusability (70, 71). Genetic data from the discovery and DANBIO populations can be accessed at ftp.genyo.es and data from the DREAM registry are available at https://www.synapse.org/#!Synapse:syn3280809/wiki/194735 and https://www.synapse.org/#!Synapse:syn3280809/wiki/194736.

## Ethics Statement

The study was approved by the ethical review committee of participant institutions: Virgen de las Nieves University Hospital (2012/89); Santa Maria Hospital-CHLN (CE 877/121.2012); University Clinical Hospital of Santiago de Compostela (2013/156); Wroclaw Medical University (KB-625/2016); Radboud university medical center (2011/299) and by the Regional Ethics Committee of Central Denmark Region (S-20120113). The patients/participants provided their written informed consent to participate in this study. 

## Author Contributions

RC and JSa designed the study and drafted the manuscript. JMSM, AM-D, and AJCS were responsible for genotyping. MALN, HC, LQ, SS, BG, MH, IF, EP-P, PC-Z, JSw, AB, SV, EP, MF, AE, MC, VA, JF, MJ, KBK, EC, and JSa coordinated the sample collection and HC, IF, and MF were involved in the records review and data acquisition. JMSM and JSa performed data quality control of genetic data and statistical analysis. MN, RTH, YL provided the functional raw data and JSa performed the analysis of functional data. All authors contributed to and approved the final version of the manuscript.

## Funding

This study was supported by grants PI17/02276 and PI20/01845 from Fondo de Investigaciones Sanitarias (Madrid, Spain) and by intramural funds of GENYO and FIBAO foundation (Granada, Spain). This study was also supported by the Novo Nordisk Fonden (NNF15OC0016932, VA) and Knud og Edith Eriksens Mindefond (VA) and Gigtforeningen (A2037, A3570, VA). JS and KB-K were supported by the grant No. 2016/21/B/NZ5/01901 from the National Science Centre (Poland). MGN was supported by a Spinoza grant from the Netherlands Organization for Scientific Research. YL was supported by an ERC Starting Grant (948207) and the Radboud University Medical Centre Hypatia Grant (2018) for Scientific Research. The funders had no role in study design, data collection and analysis, decision to publish, or preparation of the manuscript.

## Conflict of Interest

VA has received compensation for consultancy and for being a member of an advisory board from MSD (Merck) and Janssen. BG received funding for research from AbbVie, Biogen, and Pfizer. MH received funding for research from Abbvie, Biogen, BMS, CellTrion, MSD, Novartis, Orion, Pfizer, Samsung and UCB. JF received unrestricted research grants or acted as a speaker for Abbvie, Ache, Amgen, Biogen, BMS, Janssen, Lilly, MSD, Novartis, Pfizer, Roche, UCB. AB has received congress invitations, personal fees and research fees (to the department) from Boehringer, Amgen, Abbvie, Biogen, Cellgene, Pfizer, Novartis, Galapagos, Gilead, Roche, Sanofi.

The remaining authors declare that the research was conducted in the absence of any commercial or financial relationships that could be construed as a potential conflict of interest.

## Publisher’s Note

All claims expressed in this article are solely those of the authors and do not necessarily represent those of their affiliated organizations, or those of the publisher, the editors and the reviewers. Any product that may be evaluated in this article, or claim that may be made by its manufacturer, is not guaranteed or endorsed by the publisher.
