# Supplementary material for: Validation of GWAS-Identified Variants for Anti-TNF Drug Response in Rheumatoid Arthritis: A Meta-Analysis of Two Large Cohorts
Source: Front Immunol. 2021 Oct 27;12:672255. doi: 10.3389/fimmu.2021.672255 (PMC8579100; doi:10.3389/fimmu.2021.672255)
Supplement: Supplementary file 1 [file Table_1.docx]

**Supplementary Table 1.** Demographic and clinical characteristics of RA patients from the DREAM registry.

| **Anti-TNF patients (n=706)** | | | |
| --- | --- | --- | --- |
|  |  |  |  |
| *Demographic characteristics* | *DREAM registry*  *(n=706)* |  |  |
|  |  |  |  |
| *Age (years)* | 54.59 ± 12.78 |  |  |
| *Sex ratio (female/male)* | 2.07 (476/228) |  |  |
|  |  |  |  |
| *Clinical assessment* |  |  |  |
|  |  |  |  |
| *Percentage of patients with RF positivity* ^Ϯ^ | 532 (77.55) |  |  |
| *Percentage of ACPA-positive patients* ^φ^ | 150 (62.24) |  |  |
| *DAS28 at baseline* | 5.33 ± 1.26 |  |  |
| *Disease duration (years)* | 9.70 ± 9.57 |  |  |
|  |  |  |  |
| *Treatments* |  |  |  |
|  |  |  |  |
| *First biologic agent* |  |  |  |
| *Infliximab (%)* | 243 (34.41) |  |  |
| *Etanercept (%)* | 130 (18.41) |  |  |
| *Adalimumab (%)* | 333 (47.17) |  |  |
| *Golimumab (%)* | - |  |  |
| *Certolizumab (%)* | - |  |  |
| *Biosimilar Infliximab (%)* | - |  |  |
|  |  |  |  |

^Ϯ^ RF status was available for 686 patients.

^φ^ ACPA status was available for 241 patients.

Data are means ± standard deviation or n (%). Abbreviations: RF, rheumatoid factor; ACPA: anti-citrullinated protein antibodies; DAS28, disease activity score; DMARDs, disease-modifying anti-rheumatic drugs.

* Clinical data available in 708 patients (those used to genotype the most interesting markers).

RF and ACPA data were available in 598 and 527 patients in the discovery population.

RF and ACPA data were available in 688 and 243 patients in the DREAM population.

RF and ACPA data were available in 280 and 431 patients in the DANBIO population.
